# Evaluation of the Effects of Different *Bacteroides vulgatus* Strains against DSS-Induced Colitis

**DOI:** 10.1155/2021/9117805

**Published:** 2021-05-29

**Authors:** Sijia Li, Chen Wang, Chengcheng Zhang, Yanhong Luo, Qianqian Cheng, Leilei Yu, Zhen Sun

**Affiliations:** ^1^School of Food Science and Technology, Jiangnan University, Wuxi, Jiangsu 214122, China; ^2^State Key Laboratory of Food Science and Technology, Jiangnan University, Wuxi, Jiangsu 214122, China

## Abstract

Although the strain-dependent effects of *Bacteroides vulgatus* on alleviating intestinal inflammatory diseases have been demonstrated, the literature has rarely focused on the underlying causes of this effect. In this study, we selected four *B. vulgatus* strains (FTJS5K1, FTJS7K1, FSDTA11B14, and FSDLZ51K1) with different genomic characteristics and evaluated their protective roles against dextran sulfate sodium- (DSS-) induced colitis. Compared to the other three tested strains, *B. vulgatus* 7K1 more strongly ameliorated the DSS-induced weight loss, shortening of the colon length, increased disease activity index scores, colonic tissue injury, and immunomodulatory disorder. In contrast, *B. vulgatus* 51K1 significantly worsened the DSS-induced alterations in the tumor necrosis factor-alpha (TNF-*α*) concentration and colonic histopathology. A comparative genomic analysis of *B. vulgatus* 7K1 and 51K1 showed that the beneficial effects of *B. vulgatus* 7K1 may be associated with some of its specific genes involved in the production of short-chain fatty acids or capsular polysaccharides and enhancement of its survivability in the gut. In conclusion, these findings indicate that the supplementation of *B. vulgatus* 7K1 is a potentially efficacious intervention for alleviating colitis and provides scientific support for the screening of probiotics with anticolitis effect.

## 1. Introduction

*Bacteroides*, one of the most abundant genera in the mammalian colon, has been a primary candidate for next-generation probiotics and has attracted considerable attention due to its role in the prevention of a series of metabolic disorders, including obesity [1, 2], diarrhea [3], viral encephalitis [4], and enteritis [5]. In particular, the protective effects of *Bacteroides* on inflammatory diseases in the gut are a hot topic. The results of human studies have indicated that the relative abundance of *Bacteroides* in patients with inflammatory bowel disease (IBD) is markedly lower than that in healthy participants [6, 7]. Furthermore, animal studies related to colitis have demonstrated that several strains of *Bacteroides*, including *Bacteroides fragilis* NCTC 9343, *Bacteroides thetaiotaomicron* DSM 2079, and *Bacteroides cellulosilyticus* DSM 14838, can expand the population of interleukin- (IL-) 10-producing CD4^+^CD45RB^low^ T cells [8], ameliorate the histopathological damage of the gut [9], and increase the levels of anti-inflammatory IL-10 and Treg cells [10]. These findings indicate that *Bacteroides* strains could be beneficial to the restoration of gut health in patients with intestinal inflammatory diseases. However, some studies have shown inconsistent results. One study found that the oral administration of enterotoxigenic *B. fragilis* 86-5443-2-2 isolated from piglets induced colitis in mice, which was manifested by the severe damage of colon tissue [11]. In addition, *Bacteroides eggerthii* 12986 has been reported to reduce survival, accelerate body weight loss, and increase intestinal bleeding in dextran sulfate sodium- (DSS-) treated mice, which then enhanced the severity of their colitis [12]. These reports suggest that the effects of *Bacteroides* on intestinal inflammatory diseases are species or even strain-specific.

The varying effects of different strains may be attributable to their physiological characteristics. The colonization ability of strains is a physiological characteristic related to the protective functions of some strains against intestinal inflammatory disease. The type VI secretion system (T6SS) [13], antibacterial proteins [14], or capsular polysaccharides [15] of certain *Bacteroides* strains may increase their competitive fitness in the gut. Colitis-related studies have indicated that a high competitive colonization ability of *B. fragilis* could inhibit the intestinal adhesion of and further exposure to toxic pathogenic bacteria, and thus, prevent colitis [16]. The compounds produced by various strains also play a role in the development of intestinal inflammatory disease. For example, short-chain fatty acids (SCFAs), especially butyrate, may promote the intestinal epithelial barrier function [17], inhibit the central regulator of the inflammatory NF-*κ*B signaling pathway [18], and decrease oxidative stress [19], thereby preventing pathological damage of the colon associated with intestinal inflammatory disease. One study found that the administration of *B. fragilis* could improve the tight junction (TJ) integrity of the gut by increasing the number of SCFAs [20]. However, enterotoxins secreted by *B. fragilis* VPI 13784 have been reported to induce inflammation and significant colon tissue damage in lambs, rabbits, and rats after injection of the strain into their intestinal ligated loops [21]. The surface antigens of some strains can also affect intestinal inflammatory diseases. For example, polysaccharide A, a type of capsular polysaccharide present on *B. fragilis* NCTC 9343, has been reported to alleviate colitis [22] and colitis-associated colorectal cancer [23]. Another capsular zwitterionic polysaccharide TP2 from *B. fragilis* ZY-312 has been reported to protect rats from 2,4-dinitrobenzenesulfonic acid-triggered enteritis by reducing the degree of intestinal adhesion and the area of intestinal ulcers [5]. Moreover, the sphingolipids of *B. fragilis* NCTC9343 have been found to attenuate oxazolone-induced experimental colitis [24]. These results indicate that the effects of *Bacteroides* and even probiotics on alleviating intestinal inflammatory diseases are closely related to their physiological characteristics. Notably, the phenotype of bacteria is determined by their genome, and some studies have revealed that the functional differences between various probiotic strains on colitis remission are strongly associated with their genome [25, 26].

*Bacteroides vulgatus* is a representative species of the *Bacteroides* genus and is known to have a beneficial effect on the human colon health [27, 28]. Some studies have reported that *B. vulgatus* mpk can inhibit *Escherichia coli*-induced colitis [29] or *Yersinia enterocolitica*-induced inflammation [30], whereas other studies have demonstrated the proinflammatory effect of certain *B. vulgatus* strains. One study showed that *B. vulgatus* DESEP-B could induce colitis in HLA-B27 transgenic rats [31]. Another study found that *B. vulgatus* TUSVM 40G2-33 led to the enhancement of carrageenan-induced colitis in guinea pigs [32]. These results imply that the protective effect of *B. vulgatus* is strain-dependent. Furthermore, a previous study has revealed the considerable differences in inflammatory responses of guinea pigs administrated with seven different *B. vulgatus* strains in an experimental model for ulcerative colitis [33]. This demonstrated the variable ability of various *B. vulgatus* strains in the enhancement of colitis. However, most investigations of the effect of *B. vulgatus* on colitis have only focused on a single strain. Hence, it is important to investigate the complex relationship between *B. vulgatus* strains and colitis and the reasons for the varying effects of different strains. In this study, we selected four *B. vulgatus* strains with large differences in their genomes and assessed their roles in alleviating colitis. Due to its simplicity, reliability, and applicability, we used DSS to induce colitis in mice [34]. We then analyzed the genomic characteristics of the selected *B. vulgatus* strains to identify the functional genes that may play a role in alleviating the intestinal damage caused by the DSS.

## 2. Materials and Methods

### 2.1. Bacterial Strains and Preparation

We used *B. vulgatus* strains FTJS5K1 (5K1), FTJS7K1 (7K1), FSDTA11B14 (11B14), and FSDLZ51K1 (51K1) in this study, all of which had been isolated from the fecal samples of different volunteers. The 5K1 and 7K1 strains were deposited in the Culture Collection of Food Microorganisms (CCFM) of Jiangnan University (Wuxi, China). The four strains were grown anaerobically at 37°C for 18 h in a brain–heart infusion broth (Hopebio, China) supplemented with 5 *μ*g/mL hemin (Sangon Biotech, China) and 0.5 *μ*g/mL vitamin K1 (Sangon Biotech, China). A fresh culture of each strain was collected by centrifugation (5 min at 6000 g) and then washed twice with sterile phosphate-buffered saline (PBS). The final bacterial pellets were resuspended in sterile PBS at a concentration of 5 × 10^9^ colony-forming units (CFUs)/mL.

### 2.2. Animal Experimental Design

Sixty specific pathogen-free C57BL/6J mice (male, 6 weeks old) purchased from the Shanghai Laboratory Animal Center were housed at five animals per cage at the Animal Experiment Center of Jiangnan University. The mice were provided with sufficient sterilized water and standard food ad libitum and were maintained under standard conditions (20–24°C, 50%–60% humidity, and a 12 h light/darkness cycle). All mice were given a 7-day period to acclimatize to their new environment. Then, they were randomly divided into six groups (10 mice in each group): control, DSS, DSS+5K1, DSS+7K1, DSS+51K1, and DSS+11B14. To induce acute colitis in the mice in the experimental groups, 3% DSS (36–50 kDa, MP Biomedicals, Carlsbad, CA, USA) was added to their sterile filtered drinking water for 7 days. During the trial, the mice in the control and DSS groups were orally gavaged with 0.2 mL of sterile PBS. The mice in the other groups were fed one of the four *B. vulgatus* strains at a dose of 1 × 10^9^ CFUs/0.2 mL in sterile PBS by gavage. Three essential parameters of the disease activity index (DAI) were measured daily [35], including body weight, stool consistency, and fecal blood. Fecal occult blood was measured by using an Occult Blood Kit (Nanjing Jiancheng Co., Ltd., Nanjing, China). On day 7 after treatment, fresh stool samples were collected and instantly frozen at -80°C for further analysis. On day 8, euthanasia of mice was performed by carbon dioxide administration. The colons of all the mice were extracted, and their lengths were measured. The distal colon (5 mm) was then immersed in a 4% paraformaldehyde solution, and the remainder was stored at −80°C for subsequent testing. All the procedures related to these animal experiments were approved by the Committee of Ethics in Jiangnan University, China (JN. NO. 20190930c0801120[249]).

### 2.3. Genome Sequencing, Clusters of Orthologous Group (COG) Annotation, and Phylogenetic Tree Construction

Genome sequencing of *B. vulgatus* strains was performed using the Illumina HiSeq System by Majorbio (China), as described in a previous study [36, 37]. GLIMMER software was used to predict the protein-coding sequences. To identify the relationships between different *B. vulgatus* strains, we used OrthoMCL1.4 to generate the orthologous genes of 14 strains. Among these 14 strains, the genome of ATCC 8482 (Genome accession number: NC_009614.1), mpK (Genome accession number: CP013020.1), PC510 (Genome accession number: NZ_ADKO00000000.1), AF34-15 (Genome accession number: NZ_QRPW00000000.1), AM44-21 (Genome accession number: NZ_QSEZ00000000.1), RH 1270 (Genome accession number: NZ_WCIG00000000.1), and RJ2H1 (Genome accession number: NZ_PHJG00000000.1) was downloaded from the National Center for Biotechnology Information (NCBI) database. The other 7 genome sequences of 5K1 (Genome accession number: JACBPX000000000), 7K1 (Genome accession number: JACBPY000000000), 11B14 (Genome accession number: JACBPW000000000), 51K1 (Genome accession number: JACBPV000000000), FBJS10K3 (Genome accession number: JACBPS000000000), FGSZY37K4 (Genome accession number: JACBPT000000000), and FJSWX62K35 (Genome accession number: JACBPU000000000) were from the current study. We then used PhyML3 software to construct a neighbor-joining phylogenetic tree based on the core gene alignment generated using MAFFT [38]. To distinguish the functional genes between the various strains, we annotated the gene functions against the Carbohydrate-Active enZyme (CAZy) database and the Clusters of Orthologous Groups (COG) protein database by BLASTp [39].

### 2.4. Determination of Intestinal Permeability

To assess the intestinal permeability of the mice, we used 4000-Da fluorescein isothiocyanate-dextran (DX-4000-FITC, Sigma-Aldrich, USA), as described in [40]. Briefly, the mice were orally administrated DX-4000-FITC at a dosage of 500 mg/kg of body weight after fasting for 6 h on day 7. After 1 h, their blood was collected to detect the concentration of DX-4000-FITC.

### 2.5. Histological Evaluation

The fixed colon tissues were embedded in paraffin, stained with hematoxylin and eosin, and finally scanned by a Digital Slide Scanner (Motic China Group Ltd.). The damage severity of colon section was evaluated and scored from 0 to 4 for ulceration of epithelium, crypt damage, depletion of goblet cells, edema, and inflammation by a pathologist in blinded fashion.

### 2.6. Biochemical Analysis of the Colon

Colon samples of a certain weight were homogenized in normal saline solution and then centrifuged at 12000 g (10 min at 4°C). The supernatant was used to determine the total protein concentration using a BCA protein assay kit (Beyotime Biotechnology, Shanghai, China). The contents of IL-6, IL-10, and TNF-*α* in the colon supernatant were determined using commercially available enzyme-linked immunosorbent assay kits (R&D Systems, Minneapolis, MN, USA).

### 2.7. Gene Expression Analysis

Total RNA isolation from the colon tissue was performed using a FastPure Cell/Tissue Total RNA Isolation Kit (Vazyme Biotech Co., Ltd., Nanjing, China), and then a RevertAid First Strand cDNA Synthesis Kit (Vazyme Biotech Co., Ltd., Nanjing, China) was used for cDNA synthesis. Real-time quantitative polymerase chain reaction (RT-qPCR) was performed using *β*-actin as an internal control to identify the expressions of mucin2 (MUC2), ZO-1, claudin-1, and occludin [41]. RT-qPCR was carried out on a CFX96 Real-Time System (Bio-Rad, Hercules, CA) using SYBR Green super mix (Bio-Rad, Hercules, CA) under the following program: 2 min at 95°C, 39 cycles of 30 s at 95°C, 30 s at 60°C, and 30 s at 72°C. The 2^-∆∆Cq^ method was used to analyze the results.  Table 1 lists the sequences of primers used in this study.

### 2.8. Fecal DNA Extraction, Sequencing, and Analysis

The FastDNA Spin Kit for Feces (MP Biomedicals) was used to extract bacterial DNA from the stool samples of the mice. The gut microbiota genomes were sequenced according to the method described in a previous study [42]. Briefly, after amplification and purification, the DNA amplicons of the 16S rRNA sequences (V3-V4 region) in the bacterial DNA were sequenced by the MiSeq Illumina platform.

Principle coordinate analysis was performed to evaluate the beta diversity of the microbial communities. Microbial diversity was estimated by the Chao-1 index. LEfSe analysis was used to determine the intergroup differences in the fecal microbiota composition.

### 2.9. Determination of Short-Chain Fatty Acids (SCFAs) in Feces

To extract SCFAs (acetate, propionate, isobutyrate, butyrate, valerate, and isovalerate), the fecal samples were weighed, then soaked in saturated NaCl solution, acidified with sulfuric acid (10%), and treated with diethyl ether. Gas chromatography-mass spectrometry (GC-MS) was then performed to determine the SCFAs concentrations in the feces, as described in [43]. Briefly, helium was used as the carrier gas with a flow rate of 2 mL/min, and injection volume was 1 *μ*L at an injection temperature of 240°C. The following GC-MS temperature program was used: initial temperature 100°C, increase to 140°C at 7.5°C/min, then rise to 200°C at 60°C/min with a hold time of 3 min and an ion source temperature of 220°C. The external standard method was used to calculate the SCFA concentrations.

### 2.10. Statistical Analysis

All statistical analyses were performed using GraphPad Prism software version 6.0. The experimental data are expressed as the mean ± the standard error of the mean. A one-way analysis of variance with Tukey's multiple comparison test was performed to determine the significance of the differences, and *p* < 0.05 was considered to be statistically significant. The symbol ∗ indicates that the difference between the DSS group and the treated groups is significant, with ∗, ∗∗, ∗∗∗, and ∗∗∗∗ indicating *p* < 0.05, *p* < 0.01, *p* < 0.001, and *p* < 0.0001, respectively. The symbol n.s. indicates that the difference between the DSS group and other groups has no significance.

## 3. Results

### 3.1. Genetic Diversity and Evolution of *B. vulgatus* Strains

**Figure 1:**
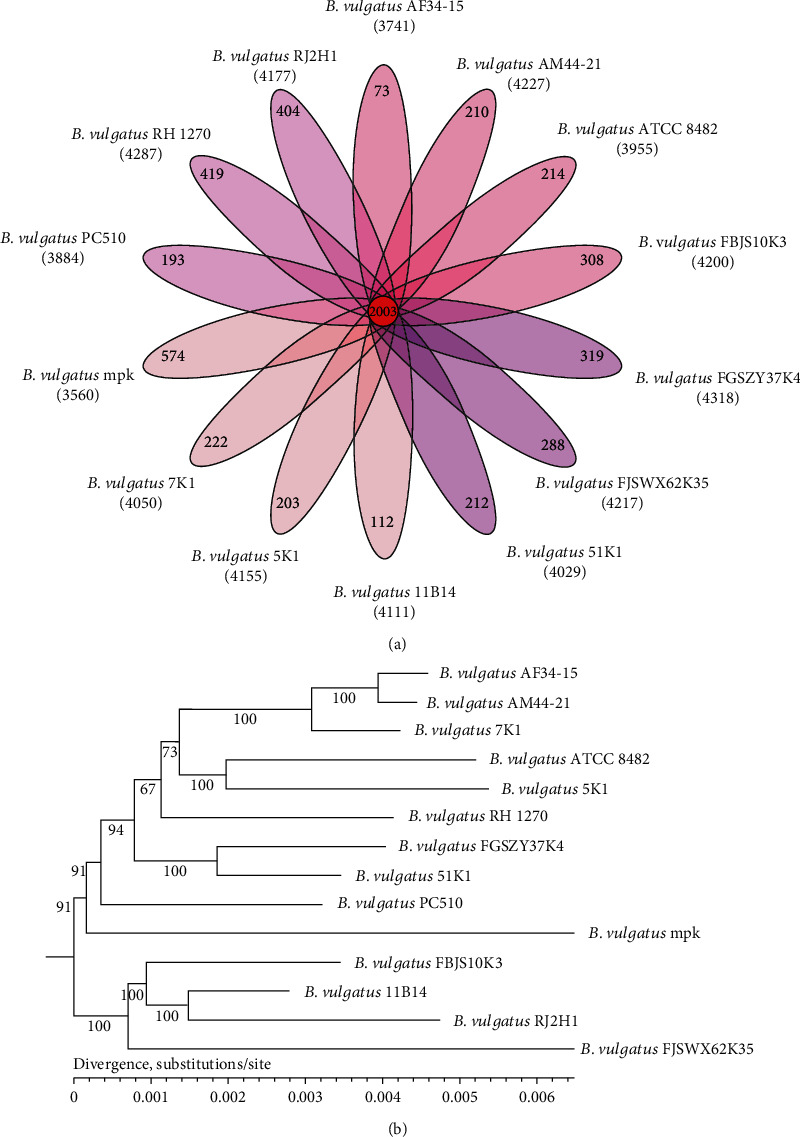
Core genes and phylogenetic analyses of 14 *Bacteroides vulgatus* strains. (a) Venn diagram of homologous clusters shared among the core genes. (b) Phylogenetic tree of 14 strains of *B. vulgatus*. Bootstrap confidence values were marked in the phylogenetic tree.

The 14 *B. vulgatus* strains (7 strains from this study and 7 strains from the NCBI database) shared 2003 orthologous genes (Figure 1(a)). The neighbor-joining tree established on the basis of these 2003 core genes shows that the strains are distributed into several branches (Figure 1(b)). We selected four strains (5K1, 7K1, 51K1, and 11B14) located far from each other on the phylogenetic tree to evaluate their effects on alleviating DSS-induced colitis in mice.

### 3.2. Effect of *B. vulgatus* on Colitis Symptoms

**Figure 2:**
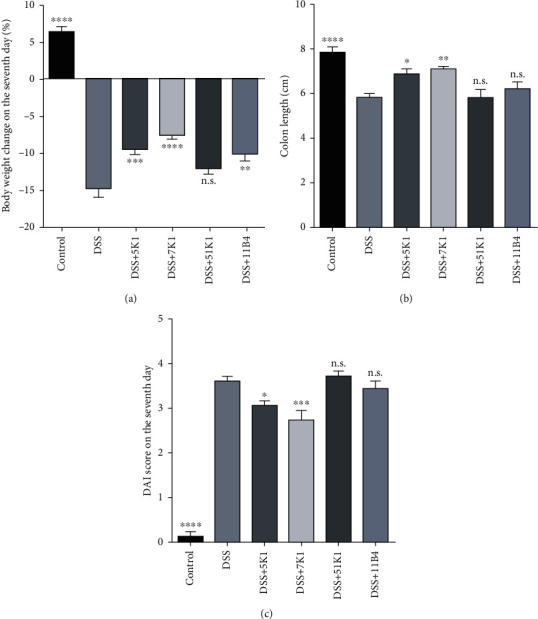
Effect of *Bacteroides vulgatus* on colitis symptoms. (a) Body weight. (b) Colon length. (c) Disease activity index (DAI). Six mice per group.

DSS exposure was found to markedly deteriorate the intestinal physiology of the mice, accompanied with weight loss, shortening of the colon length, and increased DAI scores (Figure 2). Notably, the administration of *B. vulgatus* strain 7K1, but not 5K1, 51K1, or 11B14, led to a significant recovery of these three physiological indicators.

### 3.3. Effect of *B. vulgatus* on the Intestinal Permeability of DSS-Treated Mice

**Figure 3:**
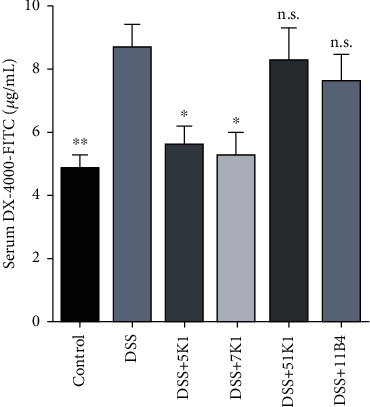
Effects of *Bacteroides vulgatus* on the intestinal permeability of mice. Four mice per group.

To estimate the effect of *B. vulgatus* strains on the intestinal permeability of the mice, we determined the serum FITC levels. After the DSS challenge, the FITC levels in the mice serum were markedly increased compared with those in the control group (Figure 3). These levels were markedly reduced in the mice fed with *B. vulgatus* 5K1 or *B. vulgatus* 7K1. However, neither *B. vulgatus* 51K1 nor *B. vulgatus* 11B14 showed any intestinal protective effect.

### 3.4. Effect of *B. vulgatus* on DSS-Induced Colonic Tissue Injury

**Figure 4:**
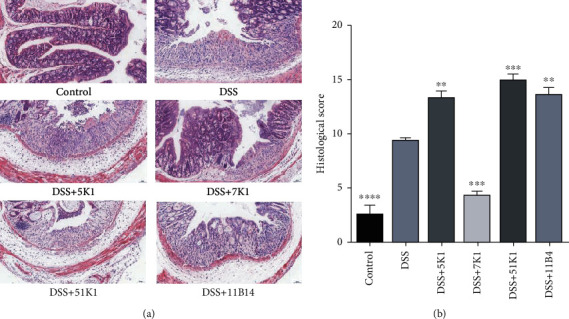
Effects of *Bacteroides vulgatus* on colonic tissue injury in mice. (a) Histological images. (b) Histological score. Four mice for the control group and three mice for each of the other groups.

The colon tissues of the mice in the DSS group, as compared to the control group, exhibited severe inflammatory cell infiltration, submucosal edema, significant disappearance of goblet cells, and severe damage to the epithelial structure (Figure 4(a)). The histological scores are a quantifiable indication of colonic injury. Compared with the DSS-treated mice, the colon tissue damage, expressed as the integrity of the intestinal epithelium and the alleviation of submucosal edema, in the mice fed with *B. vulgatus* 7K1 was significantly reduced (Figure 4(b)), whereas it was obviously aggravated in the mice fed with *B. vulgatus* 5K1, 11B14, or 51K1.

### 3.5. Effect of *B. vulgatus* on the Secretion of Inflammatory Factors in DSS-Treated Mice

**Figure 5:**
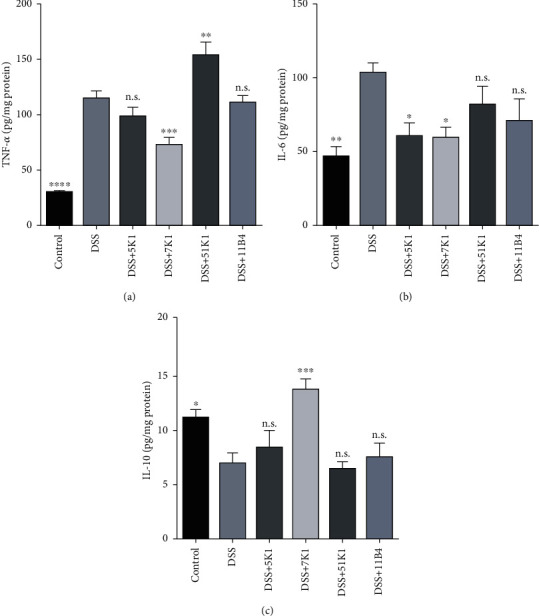
Effects of *Bacteroides vulgatus* on the concentrations of inflammatory cytokines. (a) TNF-*α*. (b) IL-6. (c) IL-10. Six mice per group.

Treatment with DSS resulted in dramatic alterations in the colonic immunomodulatory indicators of the mice, including increases in the concentrations of the proinflammatory cytokines TNF-*α* and IL-6, and a decrease in the concentration of the anti-inflammatory cytokine IL-10 (Figure 5). Among the tested strains, *B. vulgatus* 7K1 was the most effective in restoring the expression of the three inflammatory cytokines by significantly inhibiting the increases in TNF-*α* and IL-6 concentrations and upregulating the IL-10 concentrations up to those found in the control group. However, apart from reducing the IL-6 concentrations (Figure 5(b)), *B. vulgatus* 5K1 induced no alterations in any of the other indicators. In addition, *B. vulgatus* 51K1 significantly increased the TNF-*α* concentrations (Figure 5(a)).

### 3.6. Comparative Genomic Analysis of the Specific Genes in Different *B. vulgatus* Strains

We performed COG annotation to predict the functional genes of *B. vulgatus* 7K1 and *B. vulgatus* 51K1, and we found that 30 COG families were present only in the *B. vulgatus* 7K1 genome (Table S1). Except for three genes assigned to the COG category “General function prediction only” and four assigned to the COG category “Function unknown,” most of the genes were mainly related to metabolism, transport, replication, recombination, repair, and defense. In addition, according to the annotation results from the CAZy database, the abundance of genes from 14 glycoside hydrolase families (GH3, GH5, GH15, GH20, GH33, GH43_24, GH141, GH95, GH105, GH29, GH106, GH27, GH99, and GH109) and 3 glycosyl transferase families (GT28, GT6, and GT4) was relatively high in the *B. vulgatus* 7K1 genome (Table S2).

### 3.7. Effect of *B. vulgatus* on SCFA Concentrations in Feces

**Figure 6:**
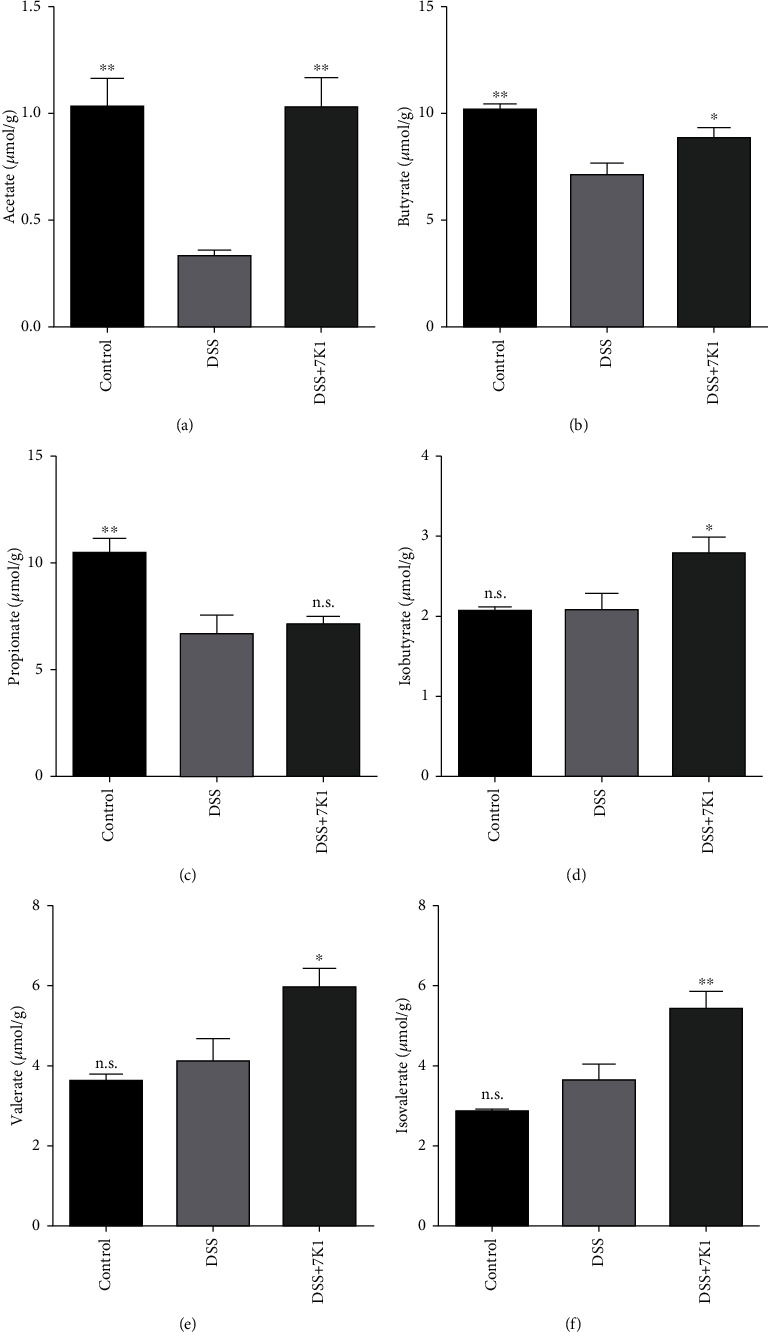
Effects of *Bacteroides vulgatus* on the concentrations of short-chain fatty acids in the fecal samples of mice. (a) Acetate. (b) Propionate. (c) Butyrate. (d) Isobutyrate. (e) Valerate. (f) Isovalerate. Five mice per group.

The concentrations of isobutyrate, valerate, and isovalerate in the fecal samples were not significantly different in the DSS group compared with the control group (Figure 6). Notably, after the oral administration of *B. vulgatus* 7K1, the concentrations of these SCFAs markedly increased. Furthermore, the concentrations of acetate, propionate, and butyrate were significantly decreased in the DSS group compared with the control group. Treatment with *B. vulgatus* 7K1 dramatically restored the acetate and butyrate concentrations close to their concentrations in the control group, but did not result in a significant change in the propionate concentrations.

### 3.8. Effect of *B. vulgatus* on the mRNA Levels of Genes Related to the Intestinal Barrier in Colon Tissue

**Figure 7:**
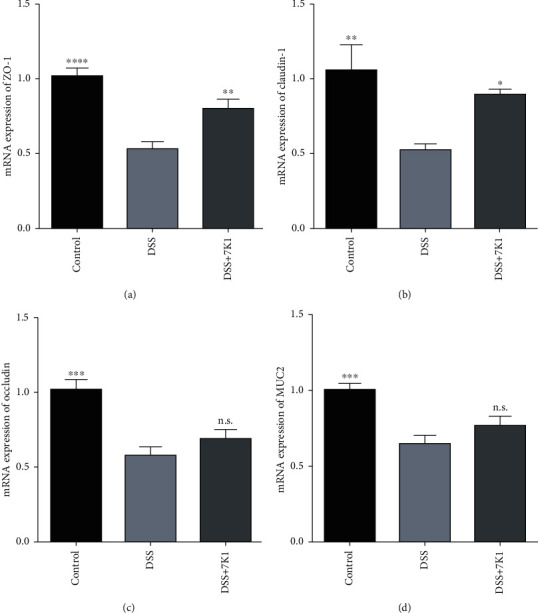
Effects of *Bacteroides vulgatus* on the intestinal barrier of mice. (a) ZO-1. (b) Claudin-1. (c) Occludin. (d) MUC2. Six mice per group.

To assess the intestinal mucosal barrier of the mice, we measured the relative expression levels of genes related to TJ proteins (ZO-1, occludin, and claudin-1) and MUC2. The results showed that the DSS treatment significantly decreased the expression of these four mucosal barrier indicators (Figure 7). Notably, oral gavage of *B. vulgatus* 7K1 played a protective role against DSS-induced alterations in ZO-1 and claudin-1 expression. Although the expression of occludin and MUC2 was upregulated by *B. vulgatus* 7K1, the results were not statistically significant.

### 3.9. Effect of *B. vulgatus* on the Composition of the Bacterial Community

**Figure 8:**
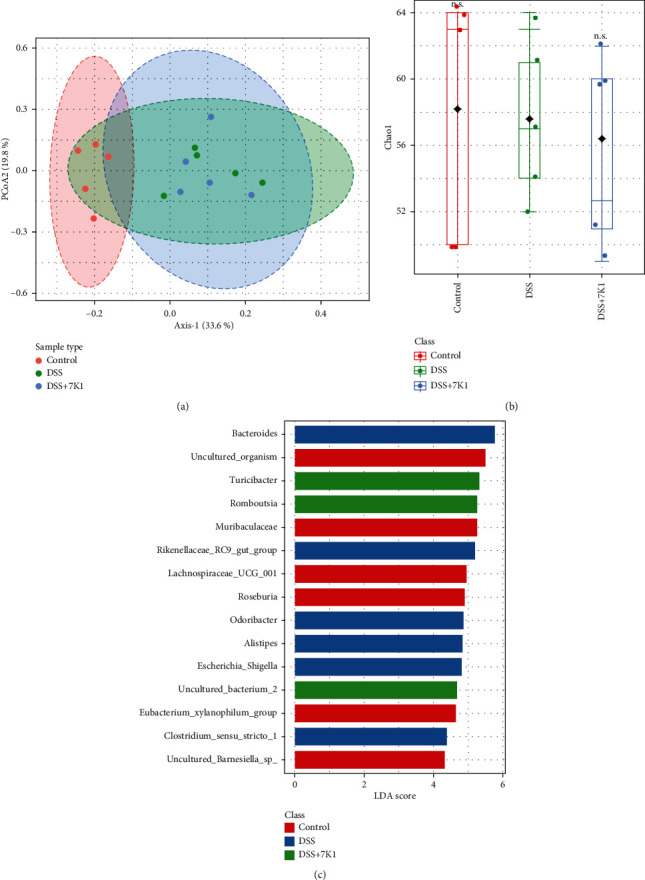
Effect of *Bacteroides vulgatus* on the intestinal microbiota of mice. (a) Principal component analysis of gut microbiota. (b) Alpha diversity indicated by Chao1 index. (c) LEfSe analysis of the different groups. Five mice per group.

Compared with the control group, DSS treatment was found to affect the composition of the gut microbiota (Figure 8(a)) and slightly decrease the microbial diversity (Figure 8(b)), although these results were not statistically significant. Cotreatments with DSS and *B. vulgatus* 7K1 induced similar results. At the genus level, *B. vulgatus* 7K1 treatment markedly increased the abundance of *Turicibacter* and *Romboutsia* in comparison with that in the DSS group (Figure 8(c)).

## 4. Discussion

Numerous studies have shown that different strains of probiotics have different anticolitis effects [44–46]. *B. vulgatus*, a next-generation probiotic, has also been found to prevent colitis, depending on the strain [33]. Many factors influence the strain-specific effects of probiotics. The survivability of probiotics during transit through the gastrointestinal tract directly affects their abundance in the gut [47]. Several studies have shown that the protective effects of probiotic strains on the host health are dose-dependent [48–50]. Research has also revealed the enhanced ability of microencapsulated *Lactobacillus rhamnosus* GG to tolerate the stomach and small intestine environments, which can strengthen its efficacy in ameliorating the symptoms of colitis [51]. Hence, the ability of probiotics to resist the harsh environment of the gastrointestinal tract is a crucial factor that influences their colitis-ameliorating effects. Pathogenic bacteria such as *Citrobacter rodentium* and enterohemorrhagic *E. coli* can attach themselves to intestinal epithelial cells and then activate an immune response in the gut that can cause severe colitis [52, 53]. Hence, the ability of probiotics to inhibit pathogen colonization in the intestine is essential to their effectiveness against colitis. A study has revealed that the oral administration of *Lactobacillus acidophilus* can decrease the colonization and translocation of *C. rodentium* and then inhibit *C. rodentium*-induced colitis [54]. In addition, some probiotics can release antimicrobial factors such as hydrogen peroxide and bacteriocins, which can kill the pathogenic bacteria or inhibit their growth [55, 56]. Enhancement of the intestinal epithelial barrier function by some probiotics is also directly related to their alleviation effect on colitis [57, 58]. SCFAs, which are produced by certain probiotics, can promote mucin expression [59] or stimulate the expression of TJ proteins [60], which serve to maintain the intestinal integrity [61]. These findings suggest that the different anticolitis abilities of probiotic strains are attributable to their complex physiological characteristics.

Genomic diversity implies functional specificity. Several *Lactobacillus fermentum* strains with considerable genomic differences have been reported to exhibit different anti-inflammatory effects on colitis in mice [46]. Hence, we constructed an evolutionary tree and selected four *B. vulgatus* strains that have large genomic differences. We then assessed their efficacy in ameliorating DSS-induced colitis in mice. Our results showed that among the four *B. vulgatus* strains, only *B. vulgatus* 7K1 could significantly relieve five DSS-induced symptoms, including reduced body weight, a shortened colon, increased DAI scores, severe damage to the colon tissue, and increased intestinal permeability. An abnormal immune response is an important indicator of the pathogenesis of colitis. Proinflammatory cytokines TNF-*α* and IL-6 have been reported to result in mucosal inflammation and aggravate immune disorders [62, 63]. Reducing TNF-*α* and IL-6 in mice with colitis was considered to be a logical target for the treatment of colitis [64]. The experiment in IL-10-deficient mice has proved the vital role of IL-10 in preventing IBD [65]. As an anti-inflammatory cytokine, IL-10 has been reported to inhibit the expression of TNF-*α* in immune regulatory processes [66]. Moreover, the protective effects of *B. fragilis* NCTC 9343 against colitis induced by trinitrobenzene sulphonic acid (TNBS) or *Helicobacter hepaticus* largely attributed to its ability to increase the production of IL-10 [8, 22]. In our study, the oral administration of *B. vulgatus* 7K1, rather than *B. vulgatus* 5K1, not only significantly reduced the concentrations of TNF-*α* and IL-6 but also markedly increased the production of IL-10 in the colon tissue of mice. Hence, given these results, we found *B. vulgatus* 7K1 to be significantly more effective in relieving DSS-induced colitis in mice than the other *B. vulgatus* strains tested in this study. We note that *B. vulgatus* 51K1 was the only one of the four strains that failed to restore the weight loss, shortened colon, and increased DAI scores caused by DSS. Furthermore, *B. vulgatus* 51K1, but not the other three strains, significantly increased both the tissue damage and TNF-*α* concentration in the mouse colons, as compared to the DSS group. These results indicate that *B. vulgatus* 51K1 can dramatically aggravate colitis.

Subsequently, we performed a comparative genomic analysis to understand the differences in the anti-inflammatory effects of *B. vulgatus* 7K1 and *B. vulgatus* 51K1. Among the 30 strain-specific COGs for *B. vulgatus* 7K1, COG2977 is involved in secondary metabolite biosynthesis, transport, and catabolism and may be related to the production of SCFAs. Moreover, GH43_24, which is more abundant in the *B. vulgatus* 7K1 genome than in the *B. vulgatus* 51K1 genome, is mainly responsible for the hydrolysis of xylan. A previous study reported that feeding mice xylooligosaccharides can increase the production of fecal SCFAs [67]. In our experiments, *B. vulgatus* 7K1 significantly promoted the production of SCFAs, including acetate, butyrate, isobutyrate, valerate, and isovalerate (Figure 6).

The protective role of SCFAs, especially butyrate, acetate, and propionate, in intestinal inflammatory diseases has been widely demonstrated and is well recognized. Mechanistically, SCFAs may promote the integrity of the intestinal epithelial barrier by increasing the synthesis of mucins in the mucosal layer [68] and of TJ proteins in the epithelial monolayer [69, 70], and thus, contribute to the remission of colitis. In addition, SCFAs attenuate colitis in mice by restoring the balance of gut microbial dysbiosis [71, 72]. In this study, feeding *B. vulgatus* 7K1 to mice protected the TJs of their intestinal epithelial cells (Figure 7), but did not restore the balance of the gut microbial dysbiosis caused by DSS (Figure 8). Hence, enhancing the integrity of the epithelial monolayer by increasing the SCFAs may be a protective mechanism of *B. vulgatus* 7K1 against DSS-induced colitis.

COG4464, which is specific to *B. vulgatus* 7K1, is responsible for the biosynthesis of capsular polysaccharides. The anti-inflammatory effect of the capsular polysaccharide produced by certain *Bacteroides* strains has been reported in several studies [5, 10]. As the best-studied zwitterionic capsular polysaccharide, polysaccharide A has been confirmed to prevent colitis by inducing the expression of IL-10 in the colon [73]. Our results showed that *B. vulgatus* 7K1, but not *B. vulgatus* 51K1, markedly upregulated the IL-10 expression in the mouse colons (Figure 5). Thus, the gene belonging to COG4464 may partly account for the anti-inflammatory property of *B. vulgatus* 7K1.

The specific genes of *B. vulgatus* 7K1, denoted as COG0270, COG1343, and COG3392, are responsible for DNA replication, recombination, and repair and are integral to cell survival [74]. In addition, another specific gene, denoted as COG0610, belonging to *B. vulgatus* 7K1 is related to the type I site-specific restriction-modification system, which has been found to protect bacteria from bacteriophage infection [75]. Bacteriophages are members of the host gut microbiota. Environmental stimuli have been reported to induce the production of infectious bacteriophages that cause lysis in their bacterial host [76]. Hence, these genes may guarantee the survival of *B. vulgatus* 7K1 in the gut of DSS-treated mice and further ensure its protective role in the host health.

Compared with *B. vulgatus* 51K1, *B. vulgatus* 7K1 has more gene copy numbers for the families GH29, GH95, and GH141. The *α*-L-fucosidases of these glycoside hydrolase families are involved in the synthesis of fucosyl-N-acetylglucosamine disaccharides [77]. It has been reported that some fucosyl-N-acetylglucosamine disaccharides inhibit the adhesion of certain enteropathogenic *E. coli* (EPEC) strains onto HT29 cells [78]. EPEC adherence onto intestinal epithelial monolayers can disrupt the barrier function [79]. Thus, the greater number of genes of these glycoside hydrolase families in *B. vulgatus* 7K1 may guarantee its protective effect on the intestinal barrier function.

## 5. Conclusion

The results of this study revealed that the protective roles of *B. vulgatus* strains selected from different clades of the phylogenetic tree against DSS-induced colitis are strain-specific. *B. vulgatus* 7K1 exhibited a significant protective effect against colitis, but *B. vulgatus* 51K1 markedly deteriorated the symptoms of colitis in mice. The results of further genomic comparisons showed that several specific genes present in the *B. vulgatus* 7K1 genome that are responsible for colonic SCFAs or capsular polysaccharide production and survival in the gut do not exist in the *B. vulgatus* 51K1 genome. This may explain the effective protection provided by *B. vulgatus* 7K1 against DSS-induced colitis, and the lack thereof by *B. vulgatus* 51K1.

## Figures and Tables

**Table 1:** Primers sequences used for RT-qPCR.

Gene	Forward	Reverse
Claudin-1	5′-GATGTGGATGGCTGTCATTG-3′	5′-CCTGGCCAAATTCATACCTG-3′
Occludin	5′-CACACTTGCTTGGGACAGAG-3′	5′-TAGCCATAGCCTCCATAGCC-3′
ZO-1	5′-CTTCTCTTGCTGGCCCTAAAC-3′	5′-TGGCTTCACTTGAGGTTTCTG-3′
Mucin 2	5′-TGCCCACCTCCTCAAAGAC-3′	5′-GTAGTTTCCGTTGGAACAGTGAA-3′
*β*-Actin	5′-GGCTGTATTCCCCTCCATCG-3′	5′-CCAGTTGGTAACAATGCCATGT-3′

## Data Availability

The data used to support the findings of this study are available from the corresponding author upon request.

## Supplementary Materials

Supplementary Materials Table S1: specific clusters of orthologous group (COG) categories in *Bacteroides vulgatus* 7K1. Table S2: details of the Carbohydrate-Active enZyme genes that are abundant in the genome of *Bacteroides vulgatus* 7K1.
