# Supplementary material for: Evaluation of the Effects of Different Bacteroides vulgatus Strains against DSS-Induced Colitis
Source: J Immunol Res. 2021 May 29;2021:9117805. doi: 10.1155/2021/9117805 (PMC8181088; doi:10.1155/2021/9117805)
Supplement: Supplementary Materials — Table S1: specific clusters of orthologous group (COG) categories in Bacteroides vulgatus 7K1. Table S2: details of the Carbohydrate-Active enZyme genes that are abundant in the genome of Bacteroides vulgatus 7K1. [file 9117805.f1.doc]

Table S3: Specific clusters of orthologous group (COG) categories in *Bacteroides vulgatus* 7K1.

| COG ID | Functional description | Functional class |
| --- | --- | --- |
| COG0270 | Site-specific DNA methylase | [L] Replication, recombination and repair |
| COG0610 | Type I site-specific restriction-modification system, R (restriction) subunit and related helicases | [V] Defense mechanisms |
| COG1146 | Ferredoxin | [F] Nucleotide transport and metabolism |
| COG1203 | Predicted helicases | [V] Defense mechanisms |
| COG1204 | Superfamily II helicase | [H] Coenzyme transport and metabolism |
| COG1343 | Uncharacterized protein predicted to be involved in DNA repair | [L] Replication, recombination and repair |
| COG1388 | FOG: LysM repeat | [M] Cell wall/membrane/envelope biogenesis |
| COG1468 | RecB family exonuclease | [V] Defense mechanisms |
| COG1518 | Uncharacterized protein predicted to be involved in DNA repair | [V] Defense mechanisms |
| COG1556 | Uncharacterized conserved protein | [C] Energy production and conversion |
| COG1887 | Putative glycosyl/glycerophosphate transferases involved in teichoic acid biosynthesis TagF/TagB/EpsJ/RodC | [MI] Cell wall/membrane/envelope biogenesis |
| COG2186 | Transcriptional regulators | [K] Transcription |
| COG2327 | Uncharacterized conserved protein | [M] Cell wall/membrane/envelope biogenesis |
| COG2361 | Uncharacterized conserved protein | [S] Function unknown |
| COG2378 | Predicted transcriptional regulator | [K] Transcription |
| COG2801 | Transposase and inactivated derivatives | [X] Mobilome: prophages, transposons |
| COG2977 | Phosphopantetheinyl transferase component of siderophore synthetase | [Q] Secondary metabolites biosynthesis, transport and catabolism |
| COG3039 | Transposase and inactivated derivatives, IS5 family | [X] Mobilome: prophages, transposons |
| COG3247 | Uncharacterized conserved protein | [S] Function unknown |
| COG3340 | Peptidase E | [E] Amino acid transport and metabolism |
| COG3392 | Adenine-specific DNA methylase | [L] Replication, recombination and repair |
| COG3410 | Uncharacterized conserved protein | [S] Function unknown |
| COG3677 | Transposase and inactivated derivatives | [X] Mobilome: prophages, transposons |
| COG4186 | Predicted phosphoesterase or phosphohydrolase | [R] General function prediction only |
| COG4249 | Uncharacterized protein containing caspase domain | [R] General function prediction only |
| COG4464 | Capsular polysaccharide biosynthesis protein | [T] Signal transduction mechanisms |
| COG4884 | Uncharacterized protein conserved in bacteria | [S] Function unknown |
| COG5017 | Uncharacterized conserved protein | [G] Carbohydrate transport and metabolism |
| COG5206 | Glycosylphosphatidylinositol transamidase (GPIT), subunit GPI8 | [O] Posttranslational modification, protein turnover, chaperones |
| COG5610 | Predicted hydrolase (HAD superfamily) | [R] General function prediction only |

Table S4: Details of the Carbohydrate-Active enZyme genes that are abundant in the genome of *Bacteroides vulgatus* 7K1.

| CAZY family | Family Known-activities |
| --- | --- |
| GH3 | β-glucosidase & xylan 1,4-β-xylosidase & β-glucosylceramidase & β-N-acetylhexosaminidase & α-L-arabinofuranosidase & glucan 1,4-β-glucosidase & isoprimeverose-producing oligoxyloglucan hydrolase & coniferin β-glucosidase & exo-1,3-1,4-glucanase & β-N-acetylglucosaminide phosphorylases & β-1,2-glucosidase & β-1,3-glucosidase & xyloglucan-specific exo-β-1,4-glucanase / exo-xyloglucanase |
| GH15 | Glucoamylase & glucodextranase & α,α-trehalase & dextran dextrinase |
| GT28 | 1,2-diacylglycerol 3-β-galactosyltransferase & 1,2-diacylglycerol 3-β-glucosyltransferase & UDP-GlcNAc: Und-PP-MurAc-pentapeptide β-N-acetylglucosaminyltransferase & digalactosyldiacylglycerol synthase |
| GH33 | sialidase or neuraminidase & trans-sialidase & anhydrosialidase & Kdo hydrolase & 2-keto-3-deoxynononic acid hydrolase / KDNase |
| GH20 | β-hexosaminidase & lacto-N-biosidase & β-1,6-N-acetylglucosaminidase & β-6-SO3-N-acetylglucosaminidase |
| GH43_24 | β-xylosidase & α-L-arabinofuranosidase & xylanase & α-1,2-L-arabinofuranosidase & exo-α-1,5-L-arabinofuranosidase & [inverting] exo-α-1,5-L-arabinanase & β-1,3-xylosidase & [inverting] exo-α-1,5-L-arabinanase & [inverting] endo-α-1,5-L-arabinanase & exo-β-1,3-galactanase & β-D-galactofuranosidase |
| GH141 | α-L-fucosidase & xylanase |
| GH95 | α-L-fucosidase & α-1,2-L-fucosidase & α-L-galactosidase |
| GH105 | unsaturated rhamnogalacturonyl hydrolase & d-4,5-unsaturated β-glucuronyl hydrolase & d-4,5-unsaturated α-galacturonidase |
| GH29 | α-L-fucosidase & α-1,3/1,4-L-fucosidase |
| GT4 | sucrose synthase & sucrose-phosphate synthase & α-glucosyltransferase & lipopolysaccharide N-acetylglucosaminyltransferase & phosphatidylinositol α-mannosyltransferase & GDP-Man: Man1GlcNAc2-PP-dolichol α-1,3-mannosyltransferase & GDP-Man: Man3GlcNAc2-PP-dolichol/Man4GlcNAc2-PP-dolichol α-1,2-mannosyltransferase & digalactosyldiacylglycerol synthase & 1,2-diacylglycerol 3-glucosyltransferase & diglucosyl diacylglycerol synthase & trehalose phosphorylase &; NDP-Glc: α-glucose α-glucosyltransferase / α,α-trehalose synthase & GDP-Man: Man2GlcNAc2-PP-dolichol α-1,6-mannosyltransferase &; UDP-GlcNAc: 2-deoxystreptamine α-N-acetylglucosaminyltransferase & UDP-GlcNAc: ribostamycin α-N-acetylglucosaminyltransferase & UDP-Gal α-galactosyltransferase & UDP-Xyl α-xylosyltransferase & UDP-GlcA α-glucuronyltransferase & UDP-Glc α-glucosyltransferase &; UDP-GalNAc: GalNAc-PP-Und α-1,3-N-acetylgalactosaminyltransferase & UDP-GalNAc: N,N'-diacetylbacillosaminyl-PP-Und α-1,3-N-acetylgalactosaminyltransferase & ADP-dependent α-maltose-1-phosphate synthase |
| GH106 | α-L-rhamnosidase & rhamnogalacturonan α-L-rhamnohydrolase |
| GH109 | α-N-acetylgalactosaminidase & β-N-acetylhexosaminidase |
| GH27 | α-galactosidase & α-N-acetylgalactosaminidase & isomalto-dextranase &β-L-arabinopyranosidase & galactan:galactan galactosyltransferase |
| GH99 | glycoprotein endo-α-1,2-mannosidase & mannan endo-1,2-α-mannanase & |
| GT6 | α-1,3-galactosyltransferase & α-1,3 N-acetylgalactosaminyltransferase & α-galactosyltransferase & globoside α-N-acetylgalactosaminyltransferase |
